# Temporal relationship between inflammation and metabolic disorders and their impact on cancer risk

**DOI:** 10.7189/jogh.14.04041

**Published:** 2024-02-16

**Authors:** Chenan Liu, Tong Liu, Qingsong Zhang, Mengmeng Song, Qi Zhang, Jinyu Shi, Li Deng, Yue Chen, Xin Zheng, Shiqi Lin, Ziwen Wang, Hailun Xie, Shuohua Chen, Shouling Wu, Hanping Shi

**Affiliations:** 1 Department of Gastrointestinal Surgery, Department of Clinical Nutrition, Beijing Shijitan Hospital, Capital Medical University, Beijing, China; 2 National Clinical Research Center for Geriatric Diseases, Xuanwu Hospital, Capital Medical University, Beijing, China; 3 Key Laboratory of Cancer FSMP for State Market Regulation, Beijing, China; 4 Laboratory for Clinical Medicine Capital Medical University, Beijing, China; 5 Department of General Surgery, Kailuan General Hospital, Tangshan, China; 6 Cardiovascular Research Institute, University of California, San Francisco, California, USA; 7 Department of Genetics, Yale University School of Medicine, New Haven, USA; 8 Department of Cardiology, Kailuan General Hospital, Tangshan, China

## Abstract

**Background:**

Inflammation and metabolic disorders are closely associated with cancer. Whether inflammation leads to metabolic disorders or vice versa during cancer initiation remains unclear. In this study, we explored this temporal relationship and the co-exposure effect on cancer risk.

**Methods:**

This prospective study had two phases. Initially, we examined the temporal relationship between inflammation (high-sensitivity C-reactive protein (CRP)) and metabolic disorders (metabolic syndrome severity Z-score (MetS-Z)) using a 3.98-year survey and cross-lagged analysis. Subsequently, we assessed the connection of co-exposure to inflammation and metabolic disorders, and the risks of overall cancer, as well as specific obesity-related, non-obesity-related, digestive system, lung, and other cancers using an 11.04-year survey and Cox proportional hazard models.

**Results:**

The cross-lagged analysis revealed that the path coefficient from baseline CRP to follow-up MetS-Z (β2 = 0.032; 95% confidence interval (CI) = 0.026, 0.046) was more significant than the path coefficient from baseline MetS-Z to follow-up CRP (β1 = 0.009; 95% CI = −0.001, 0.019). During the follow-up, 2304 cases of cancer occurred. Compared with the risk of cancer of patients with low average cumulative CRP and MetS-Z, patients with high value had a significantly increased risk (hazard ratio = 1.54, 95% CI = 1.30, 1.83). The mediation analysis showed that MetS-Z mediated the association between CRP levels and overall cancer (12.67%), digestive system cancer (10.16%), and obesity-related cancer risk (13.87%).

**Conclusions:**

Inflammation had a greater impact on metabolic disorders than vice versa. Co-exposure to inflammation and metabolic disorders significantly increased the risk of cancer, particularly digestive system and obesity-related cancers.

**Registration:**

Chinese Clinical Trial Registry: ChiCTR–TNRC–11001489.

Approximately 19.3 million new cancer cases and 10 million cancer-related deaths occur worldwide each year [1]. Cancer is a complex disease that poses a substantial threat to public health, which makes understanding its characteristics and the underlying developmental mechanisms necessary [2]. Recent studies have demonstrated a close relationship between cancer, inflammation, and metabolic disorders, with a recent report recognising ‘tumor-promoting inflammation’ and ‘deregulating cellular metabolism’ as hallmarks of cancer [3].

Inflammation is a body’s self-protective mechanism that responds to injury, infection, or other stimuli. The release of cytokines and inflammatory mediators during the inflammatory response activates immune cells, attracts them to damaged areas, and clears pathogens [4]. However, prolonged and chronic inflammation can lead to abnormal cell proliferation and genetic mutations, thereby promoting cancer development [5]. Meanwhile, metabolic disorders involve disturbances in the metabolic processes of the body, including disruption of lipid and glucose metabolism. They result in imbalances in cellular energy, increased oxidative stress, and accumulation of abnormal metabolic byproducts, negatively affecting normal cell function and homeostasis [6]. Thus, inflammation and metabolic disorders play crucial roles in the development and progression of cancer [7].

The interplay between inflammation and metabolic disorders forms a vicious cycle, where chronic inflammation promotes the development of metabolic disorders. For example, cytokines and mediators released during the inflammatory response can disrupt insulin signalling pathways, leading to insulin resistance and glucose metabolism disorders [8]. Furthermore, cytokines modulate adipose tissue differentiation and lipid metabolism, resulting in lipid abnormalities and obesity [9]. Conversely, metabolic disorders exacerbate inflammatory responses. For example, excessive consumption of a high-fat diet induces local inflammation in adipose tissue [10]. In response, the adipose tissue releases excessive fatty acids and adipokines, activates immune cells, and enhances systemic inflammatory responses [11]. However, this bidirectional relationship is akin to a rolling snowball, inflammation and metabolic disorders coexisting and mutually reinforcing, growing larger as it rolls. Despite our awareness of the damage inflammation and metabolic abnormalities can inflict on the body, it remains unclear who the initiator and precursor is in this interplay, especially in existing cohort studies [12]. Seeking and suppressing the initiator and thereby preventing the generation of this ‘snowball’ is important for cancer prevention and the improvement of public health. We hypothesised that inflammation serves as the initiating factor, driving changes in metabolic patterns and the subsequent onset of diseases.

We therefore aimed to investigate the temporal relationship between inflammation and metabolic disorders in a longitudinal prospective population-based cohort. Specifically, we investigated the association between high-sensitivity C-reactive protein (CRP) and the metabolic syndrome severity Z-score (MetS-Z) [13], and explored the combined effect of their cumulative exposure on the risk of tumour development.

## METHODS

### Study population

For this longitudinal prospective population-based cohort study, we recruited participants from the large-scale Kailuan cohort, a prospective periodic study initiated in 2006. We recruited participants by posting recruitment information at the Kailuan General Hospital and 11 affiliated community hospitals. All individuals aged ≥18 years were eligible for this study. Follow-up investigations and assessments were conducted every two years, as previously described [14]. Informed consent was obtained from all participants. The study design adhered to the principles outlined in the Declaration of Helsinki and was approved by the Ethics Committees of Kailuan General Hospital and Beijing Shijitan Hospital.

**Figure 1:**
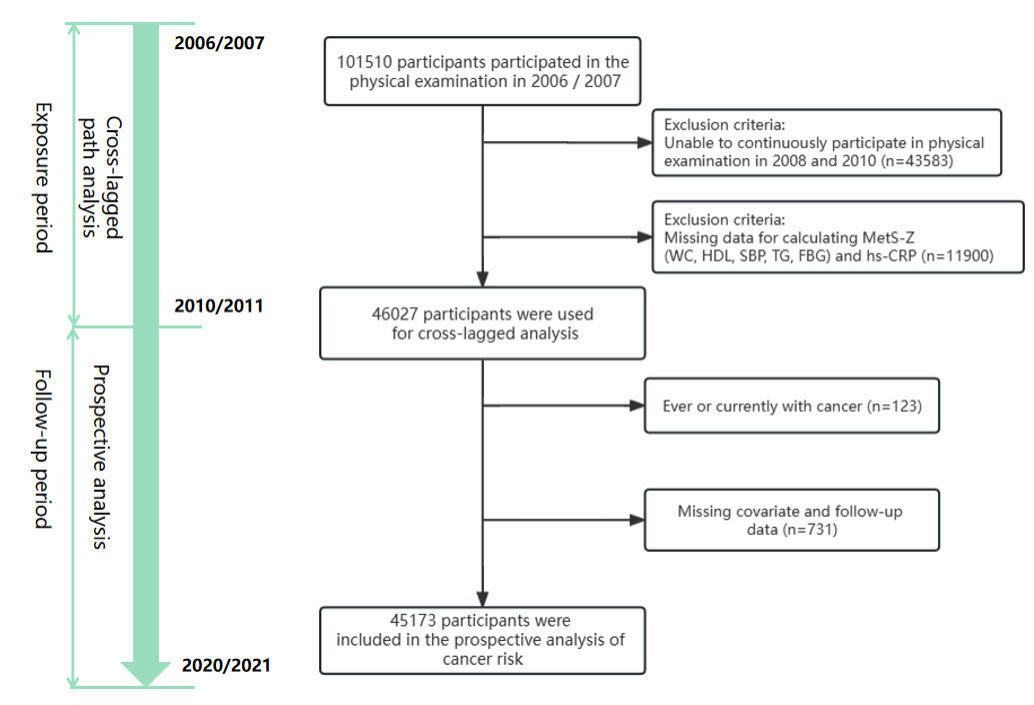
Design and flowchart of this study.

We excluded participants who did not undergo physical examinations in 2008 and 2010; those with missing information such as CRP and the variables required to calculate the MetS-Z, including waist circumference (WC) and high-density lipoprotein (HDL), systolic blood pressure (SBP), triglyceride (TG), and fasting blood glucose (Fbg) levels; those with cancer or a previous history of cancer; and those with missing covariate information. This led to a final sample of 45 173 (**Figure 1**).

### Exposures and covariates

All participants were instructed to fast for at least eight hours before visiting the hospital for blood collection. All serum samples were sent to the central laboratory at Kailuan Hospital and analysed using an automated analyser (Hitachi 747; Hitachi, Tokyo, Japan). The MetS-Z was calculated as a continuous variable using a previously validated formula (Formula S1 and Methods S1 the **Online Supplementary Document**) [13]. We calculated the average cumulative index using an algorithm based on previous studies [15], as follows:

((Index_2006/2007_ + Index_2008/2009_)/2 × time_1–2_ + (Index_2008/2009_ + Index_2010/2011_)/2 × time_2–3_)/4

Here, ‘Index_2006/2007_,’ ‘Index_2008/2009_,’ and ‘Index_2010/2011_’ refer to the CRP or MetS-Z values measured during the health examinations in 2006–07, 2008–09, and 2010–11, respectively. Meanwhile, ‘time_1–2_’ and ‘time_2–3_’ indicate the time intervals (in years) between two consecutive health examinations for each participant. We categorised the average cumulative CR*P* values into low, moderate, and high groups based on recommended Asian criteria of 1 mg/L and 3 mg/L [16], and divided the average cumulative MetS-Z values into high and low groups per a median cutoff value of 0.21. The values of these indicators measured during the exposure period are listed in Table S1 in the **Online Supplementary Document**.

The covariates were sociodemographic information (age, sex, marital status, occupation, educational level, smoking status, and alcohol consumption); anthropometric measurements (SBP, WC, and body mass index (BMI) (weight divided by height squared); and imaging or laboratory examinations (evaluations of fatty liver, CRP, Fbg, TG, total cholesterol (TC), low-density lipoprotein, and HDL levels) (Table S2 in the **Online Supplementary Document**). The questionnaire also covered sedentary time; physical activity; personal history of tumours; family history of tumours; history of hypertension and diabetes; and the use of antihypertensive, antidiabetic, and lipid-lowering medications.

### Outcomes

Our primary outcome of interest was the occurrence of cancer, which we mainly determined from International Classification of Diseases, 10th Revision codes in electronic medical records, which were further reviewed by two trained physicians based on the pathology and imaging results. Additionally, to ensure data comprehensiveness, we verified personal outcomes and the time of cancer occurrence using the Kailuan Social Security Information System, Tangshan Medical Insurance System, and the provincial cancer registration centre. Besides assessing the overall risk of cancer, we roughly categorised cancer types to ensure accessibility. Regarding the site of occurrence, we classified cancers as ‘lung,’ ‘digestive system,’ or ‘other’ cancers. Concerning aetiology, we divided cancers into obesity-related [17] and non-obesity-related risk groups (Methods S2 in the **Online Supplementary Document**). Follow-up for all participants started from the 2010–11 survey and continued until the occurrence of cancer, death, or the last follow-up date (31 December 2021).

### Statistical analyses

We presented continuous variables as means with standard deviations (SDs) or medians with interquartile ranges (IQRs) and analysed them using one-way analysis of variance or the Kruskal-Wallis test, depending on the normality of the distribution. We performed the Shapiro-Wilk test to assess the normality of data distribution, considering it to be normal if *P* > 0.05. We presented categorical variables as frequencies with percentages and compared groups using the χ^2^ test. We performed all statistical analyses in SAS software, version 9.4 (SAS Institute, Cary, NC, USA) or *R*, version 4.2.3 (R Core Team, Vienna, Austria). Unless otherwise specified, we considered a two-sided *P*-value <0.05 as statistically significant.

Our study design was broadly divided into two parts (**Figure 1**). During the exposure period (median time of 3.98 years), we conducted a cross-lagged analysis to examine the temporal relationship between inflammation and metabolic abnormalities. During the follow-up period (median time of 11.04 years), we performed a prospective analysis to investigate the risk of cancer. We then used a typical cross-lagged panel design to explore the temporal relationship between inflammation and metabolic disorders. To evaluate their causal relationship from different dimensions, we conducted cross-lagged analyses of MetS-Z and CRP, number of MetS and CRP, MetS-Z and neutral-to-lymphatic ratio (NLR), and number of MetS and NLR (Methods S1 and S3 in the **Online Supplementary Document**). We used path coefficients (β1 or β2) to indicate the directionality between the two factors and employed Fisher Z-test for examining the differences between the path coefficients derived from the standardised variables [18] to more clearly determine their causal relationship. We used Pearson correlation analysis to assess correlations between the factors. Additionally, β3 and β4 were used to represent tracking correlations, indicating the stability of the factors over time.

After establishing a causal relationship between inflammation and metabolic disorders, we conducted a mediation analysis (using the ‘mediation’ package in *R*) to examine the mediating effect of metabolic disorders on the association between inflammation and cancer risk. The reported results include the average causal mediation effects (ACME), total effects, and proportion mediated (PM) [19]. All aforementioned analyses were adjusted for baseline age; sex; educational level; marital status; smoking status, alcohol consumption; physical activity; sedentary time (Methods S4 in the **Online Supplementary Document**); family history of cancer; BMI; WC; history of hypertension and diabetes; fatty liver; and the use of antihypertensive, anti-diabetic, and lipid-lowering drugs to account for their potential influence on the results. We also conducted a joint analysis to elucidate the joint effects of average cumulative CRP (cumCRP) and MetS-Z (cumMetS-Z) on cancer risk. Here, we classified participants into different groups based on the recommended criteria for CRP levels: low, moderate, and high inflammation groups:

− G1: average cumMetS-Z below −0.21 and average cumCRP below 1 mg/L;
− G2: average cumMetS-Z equal to or above −0.21 and average cumCRP below 1 mg/L;
− G3: average cumMetS-Z below −0.21 and 1 mg/L equal to or above average cumCRP but below 3 mg/L;
− G4: average cumMetS-Z equal to or above −0.21 and 1 mg/L equal to or above average cumCRP but below 3 mg/L;
− G5: average cumMetS-Z below −0.21 and average cumCRP equal to or above than 3 mg/L;
− G6: average cumMetS-Z equal to or below −0.21 and average cumCRP equal to or above 3 mg/L).

We employed person-years to represent cancer incidence rates and Kaplan-Meier curves to depict the cumulative incidence rates of cancer in the six participant groups. A Cox proportional hazards model was used to describe the association between combined exposure to cumCRP and cumMetS-Z and cancer risk, using hazard ratios (HRs) and 95% confidence intervals (CIs) as measures. Through three models, we conducted subgroup and interaction analyses to explore the effectiveness of this association in the different strata. Model 1 represented the crude model; model 2 was adjusted for age, sex, educational level, marital status, smoking status, alcohol consumption, physical activity, sedentary time, family history of cancer, BMI, history of hypertension, history of diabetes, and fatty liver; and model 3 was adjusted for the MetS-Z components and the use of antihypertensive, antidiabetic, and lipid-lowering medications. We also performed several sensitivity analyses to assess the stability of the results, in which we excluded participants who experienced an event within one year of follow-up, those receiving medications, those with a family history of tumours, and those with acute inflammation (CRP>10 mg/L). Additionally, considering the longitudinal nature of the study, we accounted for time-dependent variables in the analysis to address potential confounding factors over time.

## RESULTS

### Cross-lagged paths and temporal relationships between inflammation and metabolic disorders

**Figure 2:**
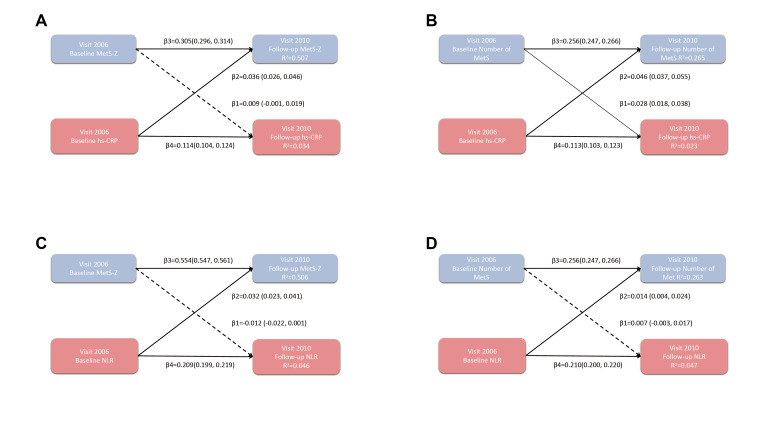
Cross-lagged path analysis of MetS (MetS-Z or number of MetS) and inflammation (CRP or NLR). To evaluate the causal relationship between inflammation and metabolic disorder from different dimensions, we conducted several cross-lagged analyses, as follows: **Panel A.** MetS-Z and CRP. **Panel B.** Number of MetS and CRP. **Panel C.** MetS-Z and NLR. **Panel D.** Number of MetS and NLR. The dashed line represents that there is no statistical difference in this path, while the solid line represents that there is a statistical difference in this path. We adjusted the cross-lagged model for age, sex, education, marital status, smoking, drinking, physical activities, sedentary, family history of cancer, BMI, waist circumference, hypertension, diabetes, fatty liver, antihypertensives, hypoglycaemic drugs, lipid-lowering drugs. Theßvalue represents the path coefficient, and R^2^ represents the variance explanation. MetS-Z – metabolic syndrome severity Z-score, CRP – high sensitivity C reactive protein, NLR – neutrophil-to-lymphocyte ratio.

We included 46 027 participants in the cross-lagged analysis. During the median exposure period of 3.98 years, after adjusting for potential confounding factors, the path coefficient from baseline CRP (2006) to follow-up MetS-Z (2010) (β2 = 0.032; 95% CI = 0.026–0.046, *P* < 0.001) was more significant than the path coefficient from baseline MetS-Z (2006) to follow-up CRP (2010) (β1 = 0.009; 95% CI = −0.001, 0.019, *P* < 0.091) (**Figure 2**, Panel A). We observed a statistically significant difference between the two coefficients (*P* < 0.001). Furthermore, the results for the other three dimensions (**Figure 2**, Panels B-D) showed similar patterns. The path coefficients from baseline inflammation to follow-up metabolic disorders were higher, indicating a stronger influence. Of note, we observed a bidirectional relationship between CRP and MetS (β1 = 0.028; 95% CI = 0.018–0.038 and β2 = 0.046; 95% CI = 0.037, 0.055), but the impact of inflammation on metabolic disorders was more pronounced (*P* < 0.001) (**Figure 2**, Panel B; Figure S1 in the **Online Supplementary Document**).

### Baseline characteristics of the combined groups

The subsequent analysis included 45 173 participants, with a mean age of 49.31 years (SD = 11.75); 79% of the participants were men (**Table 1**). Compared with the participants in the G1 group (low average cumCRP and cumMetS-Z), those in the G6 group (high average cumCRP and cumMetS-Z) were more likely to be older and smokers; have lower educational levels, a larger WC, and higher BMI and comorbidity rates; and engage in less regular physical activity. In terms of laboratory indicators, participants in the G6 group had higher MetS-Z and levels of TG, TC, Fbg, and CRP.

**Table 1:** Baseline characteristics of the study participants

		Overall	G1†	G2†	G3†	G4†	G5†	G6†
**Number of participants**	45173	8390	5458	9095	10 208	5101	6921	
**Age in years, x̄ (SD)**	49.31 (11.75)	45.40 (11.11)	46.72 (10.24)	48.34 (12.11)	49.54 (11.19)	53.59 (12.05)	53.84 (11.19)	<0.001
**Sex – men**	35 684 (79.0)	6071 (72.35)	4843 (88.73)	7091 (77.96)	8485 (83.12)	3972 (77.87)	5222 (75.45)	<0.001
**Marital status**								<0.001
Never married	677 (1.5)	170 (2.03)	70 (1.28)	203 (2.23)	127 (1.24)	63 (1.24)	44 (0.64)	
Married	43 509 (96.3)	8056 (96.02)	5303 (97.16)	8674 (95.37)	9871 (96.70)	4901 (96.08)	6704 (96.86)	
Separated or widowed	987 (2.2)	164 (1.95)	85 (1.56)	218 (2.40)	210 (2.06)	137 (2.69)	173 (2.50)	
**Work type**								0.002
Mental work	2790 (6.2)	508 (6.05)	278 (5.09)	598 (6.58)	683 (6.69)	300 (5.88)	423 (6.11)	
Manual work	42 383 (93.8)	7882 (93.95)	5180 (94.91)	8497 (93.42)	9525 (93.31)	4801 (94.12)	6498 (93.89)	
**Educational level**								<0.001
Below high school	36 728 (81.3)	6553 (78.10)	4447 (81.48)	7154 (78.66)	8300 (81.31)	4345 (85.18)	5929 (85.67)	
High School or above	8445 (18.7)	1837 (21.90)	1011 (18.52)	1941 (21.34)	1908 (18.69)	756 (14.82)	992 (14.33)	
**Sedentary time**								<0.001
<4 h	35 041 (77.6)	6162 (73.44)	4342 (79.55)	6892 (75.78)	7822 (76.63)	4223 (82.79)	5600 (80.91)	
4–8 h	8940 (19.8)	1979 (23.59)	1020 (18.69)	1933 (21.25)	2110 (20.67)	737 (14.45)	1161 (16.78)	
≥8 h	1192 (2.6)	249 (2.97)	96 (1.76)	270 (2.97)	276 (2.70)	141 (2.76)	160 (2.31)	
**Physical activity – yes**	6282 (13.9)	1192 (14.21)	627 (11.49)	1315 (14.46)	1579 (15.47)	633 (12.41)	936 (13.52)	<0.001
**Current smoker – yes**	15 412 (34.1)	2681 (31.95)	1906 (34.92)	3252 (35.76)	3730(36.54)	1526 (29.92)	2317 (33.48)	<0.001
**Current drinker – yes**	16 903 (37.4)	3304 (39.38)	2270 (41.59)	3615 (39.75)	4086 (40.03)	1525 (29.90)	2103 (30.39)	<0.001
**Family histories of cancer – yes**	1608 (3.6)	314 (3.74)	190 (3.48)	350 (3.85)	386 (3.78)	137 (2.69)	231 (3.38)	0.005
**WC, MD (IQR)**	86.0 (80.0, 93.0)	80.10 (75.00, 87.00)	87.00 (83.00, 93.00)	83.00 (77.00, 89.00)	89.20 (85.00, 95.20)	86.0 (80.00, 92.00)	92.30 (87.00, 98.50)	<0.001
**BMI, MD (IQR)**	24.93 (22.69, 27.31)	22.98 (21.09, 24.93)	25.62 (23.73, 27.60)	23.81 (21.88, 25.85)	26.47 (24.46, 28.41)	23.96 (21.97, 26.22)	26.71 (24.69, 29.04)	<0.001
**Hypertension – yes**	4833 (10.7)	400 (4.77)	497 (9.11)	631 (6.94)	1571 (15.39)	425 (8.33)	1309 (18.91)	<0.001
**Diabetes mellitus – yes**	3811 (8.4)	159 (1.90)	685 (12.55)	168 (1.85)	1479 (14.49)	133 (2.61)	1187 (17.15)	<0.001
**Fatty liver – yes**	14 691 (32.5)	952 (11.35)	2027 (37.14)	1607 (17.67)	5082 (49.78)	1106 (21.68)	3917 (56.60)	<0.001
**Antihypertensive drugs- yes**	4158 (9.2)	346 (4.12)	415 (7.60)	538 (5.92)	1354 (13.26)	357 (7.00)	1148 (16.59)	<0.001
**Hypoglycemic drugs -yes**	902 (2.0)	27 (0.32)	153 (2.80)	37 (0.41)	365 (3.58)	35 (0.69)	285 (4.12)	<0.001
**Lipid-lowering drugs - yes**	366 (0.8)	37 (0.44)	46 (0.84)	49 (0.54)	116 (1.14)	27 (0.53)	91 (1.31)	<0.001
**TG, MD (IQR)**	1.29 (0.91, 1.97)	0.93 (0.68, 1.22)	1.71 (1.22, 2.73)	1.02 (0.75, 1.33)	1.81 (1.30, 2.75)	1.00 (0.75, 1.36)	1.85 (1.33, 2.72)	<0.001
**TC, MD (IQR)**	4.91 (4.27, 5.57)	4.79 (4.20, 5.41)	4.83 (4.16, 5.52)	4.87 (4.26, 5.50)	5.01 (4.33, 5.70)	4.82 (4.22, 5.46)	5.09 (4.45, 5.76)	<0.001
**LDL, MD (IQR)**	2.33 (1.79, 2.83)	2.32 (1.85, 2.78)	2.40 (2.00, 2.81)	2.40 (1.92, 2.85)	2.40 (1.96, 2.88)	2.04 (0.71, 2.77)	2.14 (1.34, 2.84)	<0.001
**HDL, MD (IQR)**	1.51 (1.29, 1.77)	1.58 (1.36, 1.84)	1.44 (1.24, 1.69)	1.57 (1.35, 1.82)	1.42 (1.22, 1.67)	1.61 (1.38, 1.89)	1.43 (1.23, 1.68)	<0.001
**Fbg, MD (IQR)**	5.10 (4.65, 5.70)	4.93 (4.56, 5.35)	5.26 (4.80, 5.97)	4.96 (4.55, 5.40)	5.36 (4.86, 6.07)	4.84 (4.36, 5.30)	5.33 (4.80, 6.19)	<0.001
**Average CumMetS-Z, MD (IQR)**	−0.21 (−0.64, 0.25)	−0.71 (−1.03, −0.44)	0.16 (−0.05, 0.50)	−0.60 (−0.90, −0.39)	0.25 (−0.01, 0.64)	−0.62 (−0.94, −0.39)	0.36 (0.05, 0.80)	<0.001
**Average CumCRP, MD (IQR)**	1.60 (0.86, 3.18)	0.61 (0.41, 0.79)	0.66 (0.47, 0.83)	1.62 (1.27, 2.11)	1.72 (1.33, 2.23)	5.05 (3.83, 7.38)	5.18 (3.88, 7.87)	<0.001

BMI – body mass index, cumCRP – cumulative high-sensitivity C-reactive protein, cumMetS-Z – cumulative metabolic syndrome severity Z-score, Fbg – fasting blood glucose, HDL – high-density lipoprotein, IQR – interquartile range, MD – median, SD – standard deviation, TC – total cholesterol, TG – triglyceride, WC – waist circumference, x̄ – mean

*Data presented as n (%) unless specified otherwise.

†G1: average cumMetS-Z below −0.21 and average cumCRP below 1mg/L; G2: average cumMetS-Z equal to or above −0.21 and average cumCRP below 1mg/L; G3: average cumMetS-Z below −0.21 and 1mg/L equal to or above average cumCRP but below 3mg/L; G4: average cumMetS-Z equal to or above −0.21 and 1mg/L equal to or above to average cumCRP but below 3mg/L; G5: average cumMetS-Z below −0.21 and average cumCRP equal to or above 3mg/L; G6: average cumMetS-Z equal to or above −0.21 and average cumCRP equal to or above 3mg/L.

### Association between co-exposure to cumCRP and cumMetS-Z and cancer risk

**Figure 3:**
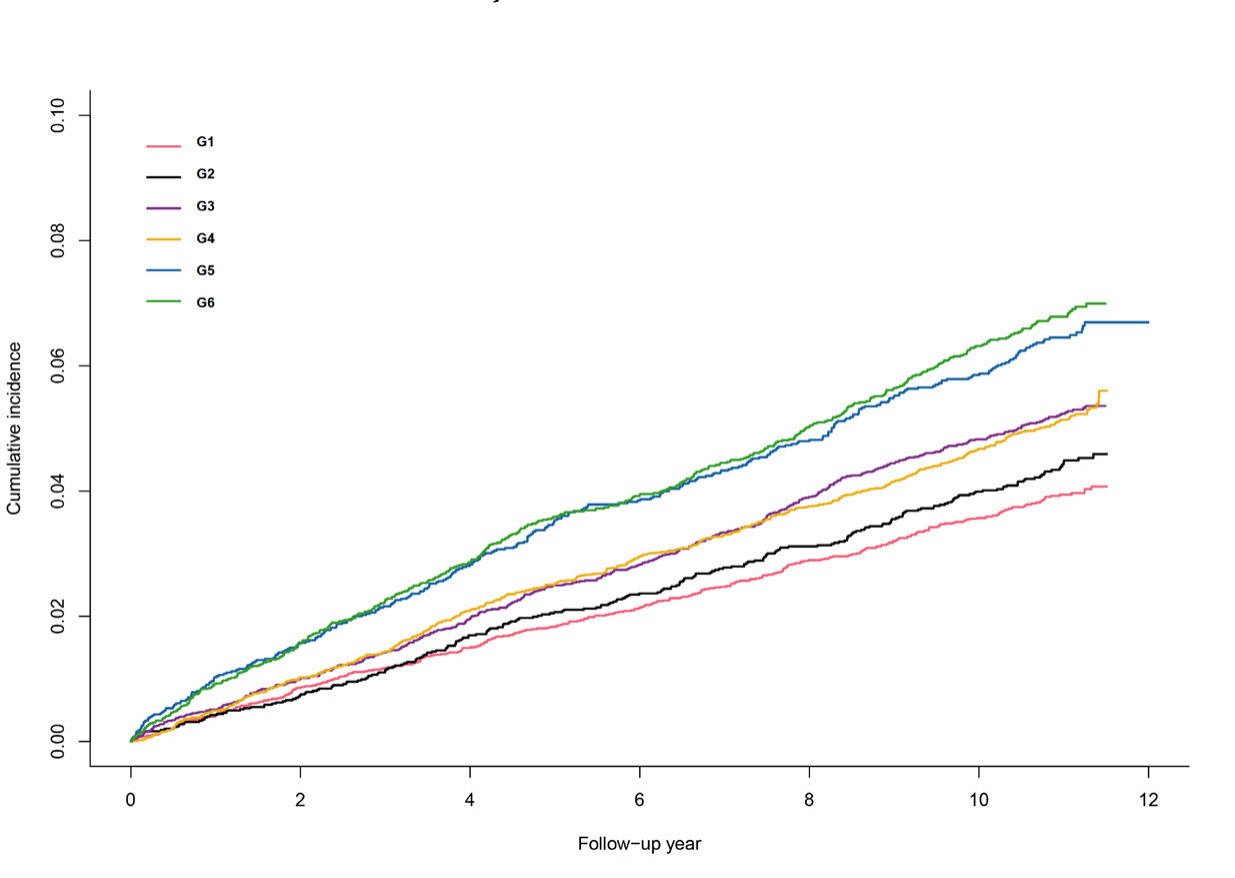
Cumulative incidence of cancer across average cumMetS-Z and average cumCRP subgroups. G1: average cumMetS-Z below −0.21 and average cumCRP below 1mg/L; G2: average cumMetS-Z equal to or above −0.21 and average cumCRP below 1mg/L; G3: average cumMetS-Z below −0.21 and 1mg/L equal to or above to average cumCRP but below 3mg/L; G4: average cumMetS-Z equal to or above −0.21 and 1mg/L equal to or above to average cumCRP but below 3mg/L; G5: average cumMetS-Z below −0.21 and average cumCRP equal to or above 3mg/L; G6: average cumMetS-Z equal to or above −0.21 and average cumCRP equal to or above 3mg/L.

We initially stratified participants based on cumMetS-Z and examined the cancer risk among individuals with different cumCRP levels. During the median follow-up period of 11.04 years (IQR = 10.66, 11.33), 2304 cancer events occurred. Irrespective of metabolic burden, an increase in the average cumCRP level was associated with an elevated overall cancer risk. Moreover, the risk increased with higher levels of inflammation (*P*-value for trend <0.05) (Table S3 in the **Online Supplementary Document**). Subsequently, we conducted a joint analysis based on the reference values of these two indicators and investigated their association with cancer risk. As the levels of cumCRP and cumMetS-Z increased, the cumulative incidence of cancer also increased (**Figure 3**). The Cox regression results (**Table 2**) showed that, after adjusting for potential confounding factors, compared with the participants in the G1 group, those in the G3 to G6 groups (i.e. those primarily characterised by elevated inflammation, with G3 to G4 representing moderate inflammation and G5 to G6 representing high inflammation) exhibited varying degrees of increased overall cancer risk (G3: HR = 1.28; 95% CI = 1.11, 1.48; G4: HR = 1.27; 95% CI = 1.09, 1.49; G5: HR = 1.45; 95% CI = 1.23; 1.71, and G6: HR = 1.54; 95% CI = 1.30, 1.83). Moreover, the G6 group showed significantly increased risks of obesity-related cancers (HR = 1.65; 95% CI = 1.37, 1.99), digestive system cancers (HR = 1.66; 95% CI = 1.26, 2.20), and lung cancer (HR = 1.62; 95% CI = 1.15, 2.27). Interestingly, there was no significant increase in the risk of non-obesity-related cancers (HR = 1.18; 95% CI = 0.80, 1.74) among participants in the G6 group.

**Table 2:** HRs for cancer risk upon co-exposure stratified by average CumCRP thresholds (1, 3 mg/L) and average CumMetS-Z (median)

	G1*	G2*	G3*	G4*	G5*	G6*
**Overall cancer**						
Events/total	327/8390	237/5458	466/9095	512/10 208	316/5101	446/6921
IR per 1000 person-years	3.65	4.10	4.83	4.88	6.13	6.45
Model 1†	ref	1.12 (0.95, 1.33)	1.32 (1.15, 1.52)	1.34 (1.16, 1.54)	1.68 (1.44, 1.96)	1.76(1.53, 2.04)
Model 2†	ref	1.09 (0.91, 1.29)	1.20 (1.03, 1.39)	1.28 (1.11, 1.47)	1.44 (1.23, 1.68)	1.44 (1.23, 1.69)
Model 3†	ref	1.15 (0.97, 1.38)	1.28 (1.11, 1.48)	1.27 (1.09, 1.49)	1.45 (1.23, 1.71)	1.54 (1.30, 1.83)
**Obesity-related cancer‡**						
Events/total	253/8390	196/5458	373/9095	430/10 208	270/5101	373/6921
IR per 1000 person-years	2.82	3.39	3.90	4.06	5.24	5.39
Model 1†	ref	1.20 (1, 1.44)	1.38 (1.18, 1.62)	1.44 (1.23, 1.68)	1.85 (1.56, 2.20)	1.91 (1.63, 2.24)
Model 2†	ref	1.14 (0.98, 1.38)	1.35 (1.15, 1.58)	1.34 (1.14, 1.58)	1.70 (1.43, 2.01)	1.71 (1.44, 2.03)
Model 3†	ref	1.22 (1.01, 1.49)	1.31 (1.12, 1.54)	1.37 (1.15, 1.63)	1.57 (1.31, 1.88)	1.65 (1.37, 1.99)
**Non obesity-related cancer**						
Events/total	74/8390	41/5458	93/9095	82/10 208	46/5101	73/6921
IR per 1000 person-years	0.83	0.71	0.97	0.77	0.89	1.06
Model 1†	ref	0.86 (0.59, 1.26)	1.18 (0.87, 1.60)	0.94 (0.68, 1.28)	1.08 (0.75, 1.56)	1.28 (0.92, 1.76)
Model 2†	ref	0.90 (0.60, 1.33)	1.18 (0.87, 1.60)	0.91 (0.65, 1.28)	1.02 (0.70, 1.48)	1.13 (0.79, 1.63)
Model 3†	ref	0.93 (0.61, 1.40)	1.18 (0.86, 1.60)	0.94 (0.66, 1.35)	1.04 (0.71, 1.53)	1.18 (0.80, 1.74)
**Digestive system cancer§**						
Events/total	107/8390	92/5458	152/9095	174/10 208	119/5101	144/6921
IR per 1000 person-years	1.94	1.59	1.59	1.64	2.31	2.08
Model 1†	ref	1.33 (1.01, 1.76)	1.33 (1.04, 1.70)	1.37 (1.08, 1.75)	1.74 (1.36, 2.24)	1.93 (1.49, 2.51)
Model 2†	ref	1.17 (0.88, 1.55)	1.19 (0.92, 1.53)	1.22 (0.96, 1.57)	1.43 (1.09, 1.88)	1.57 (1.21, 2.05)
Model 3†	ref	1.24 (0.92, 1.66)	1.24 (0.96, 1.59)	1.27 (1.01, 1.66)	1.61 (1.20, 2.16)	1.66 (1.26, 2.20)
**Lung cancer**						
Events/total	79/8390	66/5458	131/9095	131/10 208	80/5101	109/6921
IR per 1000 person-years	0.88	1.14	1.37	1.24	1.55	1.58
Model 1†	ref	1.29 (0.93, 1.80)	1.56(1.18, 2.06)	1.41 (1.06, 1.86)	1.77 (1.30, 2.41)	1.80 (1.34,2.40)
Model 2†	ref	1.29 (0.92, 1.80)	1.50(1.13, 1.98)	1.33 (1, 1.79)	1.51 (1.10, 2.07)	1.54 (1.12,2.11)
Model 3†	ref	1.4 1(1, 2.01)	1.51(1.14, 1.99)	1.45(1.06, 1.98)	1.46 (1.05, 2.02)	1.62 (1.15,2.27)
**Other cancer**						
Events/total	327/8390	79/5458	183/9095	207/10 208	117/5101	193/6921
IR per 1000 person-years	3.65	1.37	1.91	1.95	2.27	2.79
Model 1†	ref	0.87 (0.66, 1.14)	1.21 (0.98, 1.51)	1.24 (1, 1.53)	1.44 (1.12, 1.87)	1.76 (1.42,2.19)
Model 2†	ref	0.94 (0.71, 1.25)	1.21 (0.97, 1.51)	1.15 (0.91, 1.46)	1.31 (1.02, 1.68)	1.38 (1.08,1.77)
Model 3†	ref	0.98 (0.73, 1.31)	1.21 (0.96, 1.51)	1.20 (0.94, 1.54)	1.33 (1.03, 1.72)	1.45 (1.12,1.89)

IR – incidence rate, ref – reference

*G1: average cumMetS-Z below −0.21 and average cumCRP below 1mg/L; G2: average cumMetS-Z equal to or above −0.21 and average cumCRP below 1mg/L; G3: average cumMetS-Z below −0.21 and 1mg/L equal to or above average cumCRP but below 3mg/L; G4: average cumMetS-Z equal to or above −0.21 and 1mg/L equal to or above to average cumCRP but below 3mg/L; G5: average cumMetS-Z below −0.21 and average cumCRP equal to or above 3mg/L; G6: average cumMetS-Z equal to or above −0.21 and average cumCRP equal to or above 3mg/L.

†Model 1 was the crude model. Model 2 was adjusted for age, sex, education, marital status, smoking, drinking, physical activities, sedentary, family history of cancer, BMI, hypertension, diabetes, and fatty liver. Model 3 was adjusted for model 2 and antihypertensives, hypoglycaemic drugs, lipid-lowering drugs, waist circumference, HDL, SBP, TG, and FBG.

‡Obesity-related cancers include oesophagus, stomach, colon, rectum, liver, pancreas, lung, malignant melanoma, breast, corpus uteri, ovaries, prostate, kidney, bladder, brain, lymphoid and hematopoietic cancer.

§Digestive system cancers include oesophagus, stomach, small intestine, colon, rectum, liver, and pancreas cancer, as well as bile and extrahepatic cholangiocarcinoma.

Subgroup analyses revealed significant interactions between combined exposure and age (*P* = 0.001), smoking status (*P* = 0.039), fatty liver disease (*P* = 0.001), and diabetes (*P* = 0.019). The G6 group had higher risks of cancer than did the G1 group in the following subgroups: participants who were aged <60 years (HR = 1.72; 95% CI = 1.41, 2.10), non-smokers (HR = 1.53; 95% CI = 1.23, 1.89), without fatty liver (HR = 1.74; 95% CI = 1.41, 2.14), and without diabetes (HR = 1.51; 95% CI = 1.27, 1.80).

### Additional analysis and sensitivity analysis

To further elucidate the impact of the combined exposure on cancer risk, we conducted a joint analysis based on the reference cutoff values of baseline CRP and MetS-Z levels in the participants. This was performed to assess the effects of transient inflammation and exposure to metabolic dysregulation (Table S5). Similar to the overall results, participants in the G6 group had a significantly higher risk of overall cancer (HR = 1.54; 95% CI = 1.30, 1.83), obesity-related cancer (HR = 1.65; 95% CI = 1.37, 1.99), digestive system cancer (HR = 1.66; 95% CI = 1.26, 2.20), and lung cancer (HR = 1.62; 95% CI = 1.15, 2.27) than did the G1 group. However, we found no significant increase in the risk of non-obesity-related cancers.

In the sensitivity analysis, after excluding patients who experienced an event in the first year, were using medications, had a family history of cancer, had high-fat diet pattern or had acute inflammation (CRP>10 mg/L), the increased cancer risk in the G3 to G6 groups remained. The results remained robust after adjusting for time-dependent variables (Table S6 in the **Online Supplementary Document**).

### Mediation analysis

We also explored the mediating effects of MetS-Z on the association between CRP levels and risk of common cancers (Figure S2 in the **Online Supplementary Document**). The results indicated that MetS-Z mediated the association between CRP and overall cancer risk (ACME = 0.049; 95% CI = 0.031, 0.068, PM = 12.67%) and the risks of digestive system cancer (ACME = 0.038; 95% CI = 0.026, 0.050, PM = 10.16%) and obesity-related systemic tumours (ACME = 0.052; 95% CI = 0.036, 0.073, PM = 13.87%). However, we observed no significant mediating effects for lung cancer, other cancer types, and non-obesity-related cancers.

## DISCUSSION

In this study, we conducted a cross-lagged analysis of a large-scale longitudinal cohort and found a bidirectional relationship between inflammation and metabolic disorders, with inflammation having a greater impact on future metabolic disorder occurrence. The mediation analysis indicated that the MetS-Z metabolic index partially mediated the association between inflammation and overall cancer risk, and more specifically between digestive system cancer and obesity-related cancer risks. Furthermore, the analysis of the prospective cohort showed that combined exposure to inflammation and metabolic disorders exacerbated participants’ overall cancer risk or the risks of lung, digestive system, and obesity-related cancers.

Inflammation and metabolism are closely interconnected processes within the body and are characterised by a complex bidirectional regulatory relationship. Recent research has demonstrated the mutual interaction between inflammation and metabolism and their key roles in various diseases. Lee et al. [20] demonstrated that factors or kinases released by proinflammatory macrophages, such as tumor necrosis factor-α (TNF-α) and c-Jun N-terminal kinases (JNK), can exert paracrine or disruptive effects on insulin-targeted cells. They can inhibit insulin receptor signalling by inducing serine/threonine phosphorylation of insulin receptor substrates, leading to impaired insulin signalling and insulin resistance, as well as abnormal expansion and accumulation of adipocytes [21,22]. The accumulation of adipose tissue can also lead to decreased circulating levels of adiponectin (an endogenous insulin sensitiser) and anti-inflammatory factors. Adiponectin can in turn inhibit the activation of TNF-α, NF-κB, and IL-6, thereby attenuating inflammatory responses [23]. Simultaneously, the expansion of adipocytes accelerates the local accumulation of inflammatory cytokines and their elevation in systemic circulation, thereby triggering more profound inflammatory responses [24]. This is also demonstrated by Watanabe et al. [25], indicating that adipocyte is the intersection and core region of metabolism and inflammation. The accumulation of fat cells and the initiation of abnormal metabolism are triggered by the inflammatory factor IL-1β and neutrophil infiltration, mediating the activation of inflammatory bodies and the NF-κB pathway. Conversely, reactive oxygen species derived from mitochondria or the endoplasmic reticulum can also activate JNK, leading to insulin resistance and impaired feedback mechanisms in the body, resulting in increased reactive oxygen species production and more extensive inflammatory responses [26,27]. These two factors reinforce each other through a vicious cycle, causing excessive nutrient synthesis, cellular growth, homeostatic breakdown, and disease development. Our previous research also systematically showed a close relationship between adipose accumulation, inflammation, and comorbidities such as cardiovascular diseases in individuals with obesity [28]. Similarly, Dugani et al. [29] found a significant correlation between lipid, inflammatory, and metabolic biomarkers and the risk of cardiovascular disease.

However, there is limited research detailing the temporal relationship between inflammation and metabolic disorders, which we attempted to address by examining their origins. Inflammation, an adaptive response that has evolved to restore balance within the body, can be triggered by various factors, including infection, injury, cellular stress, and autoimmune processes [30]. Concerning the aetiology of metabolic abnormalities, research suggests that certain lifestyle and environmental factors such as overeating and lack of physical activity are major contributors to the development of metabolic syndrome [31]. The factors that primarily trigger metabolic dysfunction are unhealthy lifestyle habits that induce inflammation and stress responses in visceral fat, leading to increased protein breakdown, lipid oxidation, acidification, and decreased tissue responsiveness to insulin. These pathways have been shown to be important triggering factors for the activation of most pathways associated with metabolic syndrome [32,33].

In this study, we approached this issue from a clinical perspective and described the temporal relationship between inflammation and metabolic dysregulation, as well as the combined effect of their exposure on cancer risk using a prospective cohort design. Consistent with basic research findings, the results of the cross-lagged analysis also suggested that inflammation initiates metabolic disorders and diseases. The Cox regression analysis further indicated that while metabolic disorders contribute to cancer risk to some extent, the accumulation of inflammation better distinguished participants’ cancer risk (**Figure 3**, **Table 2**). Moreover, regardless of metabolic status, inflammation is a good predictor of cancer. Interestingly, when we differentiated between obesity-related and non-obesity-related cancers, there was a divergence in predictive ability. Similar to our study, Lee et al. also demonstrated that while combined exposure to systemic inflammation and insulin resistance can effectively predict cancer-related mortality in patients, they found that the predictive ability of inflammation remained regardless of insulin resistance levels [34]. Li et al. [35] showed that metabolic disorders synergistically increase the risk of colorectal cancer and abdominal obesity induced by inflammation, exerting a significant mediating effect. These findings, including both basic and clinical research, collectively suggest that inflammation is more likely to be the initiating factor for metabolic dysregulation and disease than the reverse.

Our study has several strengths. First, we derived our findings from a long-term rigorously controlled longitudinal cohort, ensuring the reliability of the data and conclusions, while overcoming the limitations of cross-sectional studies. Second, we employed an adequate exposure time and utilised a typical cross-lag analysis to determine the temporal sequence and causal relationship between inflammation and metabolic dysfunction. Third, after establishing potential causal relationships, we conducted a follow-up over a period of 11.04 years to assess the impact of cumulative exposure to both factors on cancer risk. From a clinical standpoint, our findings underscores the significance of inflammation as a pivotal factor in view of cancer prevention and treatment, as the levels of inflammation may serve as a crucial factor in assessing patient conditions. This also bears significance for public health, where stakeholders should advocate for the promotion of anti-inflammatory diets (e.g. Mediterranean diet) and exercise-focused lifestyle [36]. Furthermore, it emphasises the need to consider the detrimental effects of inflammation in the development of personalised health management and anticancer strategies.

However, our study also has some limitations. First, the community-based cohort was predominantly composed of Chinese males. Previous research suggests that women may be more susceptible to autoimmune diseases and that there are significant metabolic differences between men and women; our subgroup and sensitivity analyses likewise have limitations in addressing these factors [37,38]. Second, regarding the study design, we did not include several potential confounding factors such as types of physical activity (moderate or vigorous, work-related or recreational). Different types of physical activity may have varying degrees of resistance to inflammation and metabolic disorder [39]. Well-designed prospective studies, which should also control for confounding factors, could attempt to validate our findings and further observe the crucial role of anti-inflammatory interventions in resisting metabolic abnormalities and cancer. Third, although we used CRP, NLR, MetS-Z, and the number of MetS components as surrogate markers for inflammation and metabolic disorder, we acknowledge that inflammation and metabolic disorder are broad concepts. Despite considering factors such as WC, blood pressure, lipid levels, and Fbg, we did not comprehensively address markers of metabolic diseases like non-alcoholic fatty liver disease or gout [40]. Consequently, these may not fully reflect the levels of inflammation and the extent of metabolic disorder in the body.

## CONCLUSIONS

The relationship between inflammation and metabolism is bidirectional, making it difficult to establish a definitive causal sequence like the chicken or egg questions. This study aimed to elucidate the association between these two factors in a clinical cohort. While we observed a bidirectional relationship, the robustness of our results and the statistical differences suggest a stronger inclination toward inflammation exacerbating the increase in metabolic burden. This metabolic burden mediates the relationship between inflammation and cancer development. Combined exposure to both these factors significantly increases the risk of cancer, particularly those associated with metabolism. In practical terms, recognizing the pivotal role of inflammation in amplifying metabolic challenges has profound implications for clinical strategies. Targeting inflammation as a potential intervention point, such as adopting a sensible anti-inflammatory diet and physical activity, may help reduce the risk of cancer, especially for those cancers closely associated with inflammation and metabolic disorder.

## Additional material


Online Supplementary Document.
