# Supplementary material for: Temporal relationship between inflammation and metabolic disorders and their impact on cancer risk
Source: J Glob Health. 2024 Feb 16;14:04041. doi: 10.7189/jogh.14.04041 (PMC10869135; doi:10.7189/jogh.14.04041)
Supplement: Online Supplementary Document. [file jogh-14-04041-s001.pdf]

Supplementary Materials

Formula S1

Calculation method of Mets-Z[1]

Men：Mets-Z score= -5.4559+0.0125*WC-0.0251*HDL+0.0047*SBP+0.8244*lnTG+0.0106*FBG

Women：Mets-Z score= -7.2591+0.0254*WC-0.0120*HDL+0.0075*SBP+0.5800*lnTG+0.0203*FBG

Methods S1 According to the third report of the adult education group of the American Cholesterol Education Program (NCEPATP III) [2] ,MetS was defned based on the presence of three or more of the following components: (1) high glucose: FPG>5.6 mmol/L or diabetes diagnosis previously; (2) high blood pressure: SBP ⩾ 130 mmHg or DBP ⩾ 85 mmHg, or hypertension diagnosis previously; (3) high triglycerides: triglycerides > 1.69 mmol/L; (4) low HDL-C: HDL-C<1.04 mmol/L in men or 1.29 mmol/L in women; and (5) visceral adiposity: waist circumference⩾85 cm in men or 80 cm in women.

Methods S2 ICD10

The cancer types included were as follows: digestive system cancers [esophageal cancer (C15), gastric cancer (C16), small intestine cancer (C17), colorectal cancer (CRC; C18–C21), liver cancer (C22.0), pancreatic cancer (C25), gallbladder and extrahepatic bile duct cancer (C23–C24)], lung cancer (C34), other cancers [kidney cancer (C64–C65), bladder cancer (C67)], lymphoma (C81–C89) and leukemia (C90–C96), breast cancer (C50), cervix cancer (C53), uterus cancer (C54–C55), ovarian cancer (C56), prostate cancer (C61), and head and neck cancer (00-14, 30-32, 71, 73).

Obesity-related cancer include esophagus (C15), stomach (C16), colon (C18), rectum (C20), liver (C22), pancreas (C25), lung (C34), malignant melanoma (C43), breast (C50), corpus uteri (C54), ovaries (C56), prostate (C61), kidney (C64), bladder (C67), brain (C71) and lymphoid and hematopoietic cancer (abbreviated as “blood cancer” in the following; C81-C96), others are considered non obesity-related cancer.

Methods S3 Calculation method of NLR: NLR=Neutrophil/lymphocyte*100%.

Methods S4 Physical activity: ≥ 3 times/week, ≥ 30 minutes/time of physical exercise is considered regular physical exercise. Sedentary time: Maintain sitting position for at least 8 hours per day.

Figure S1 Heatmap of correlation between inflammatory indicators and MetS-Z

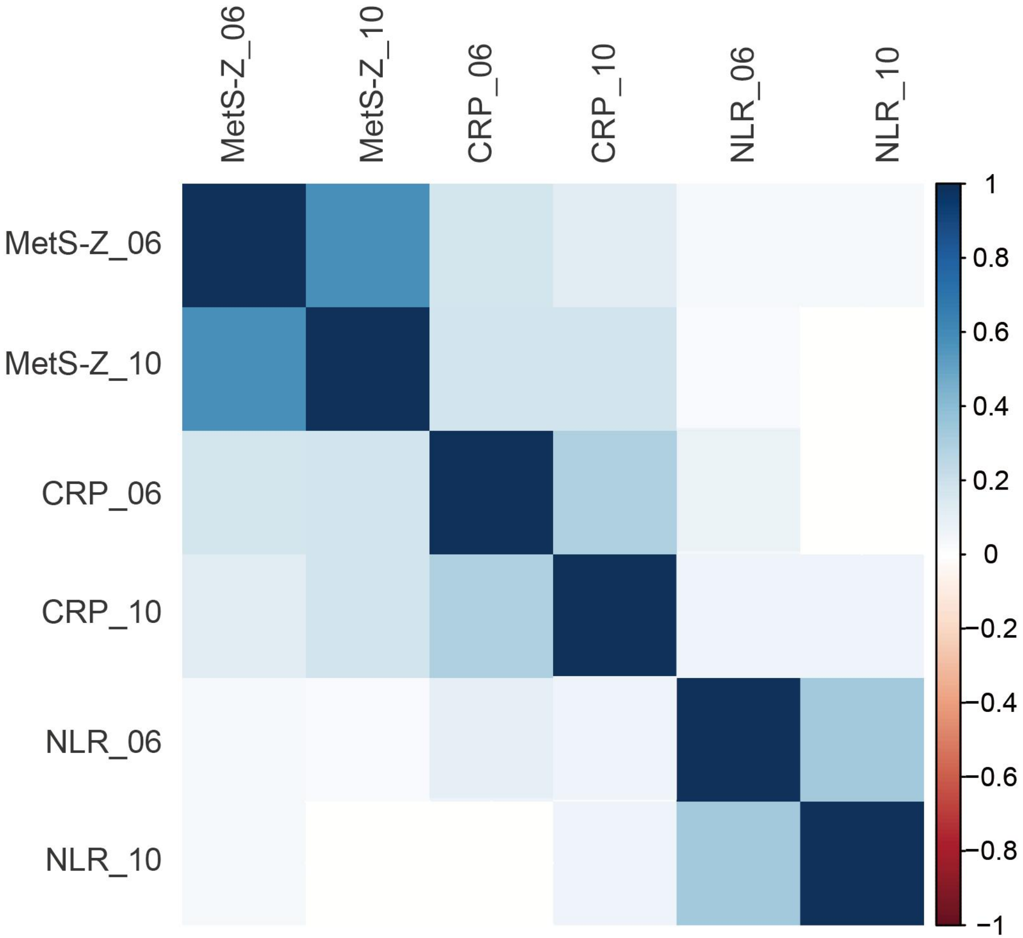

Figure S2 The mediating association between hs-CRP and cancer risk.

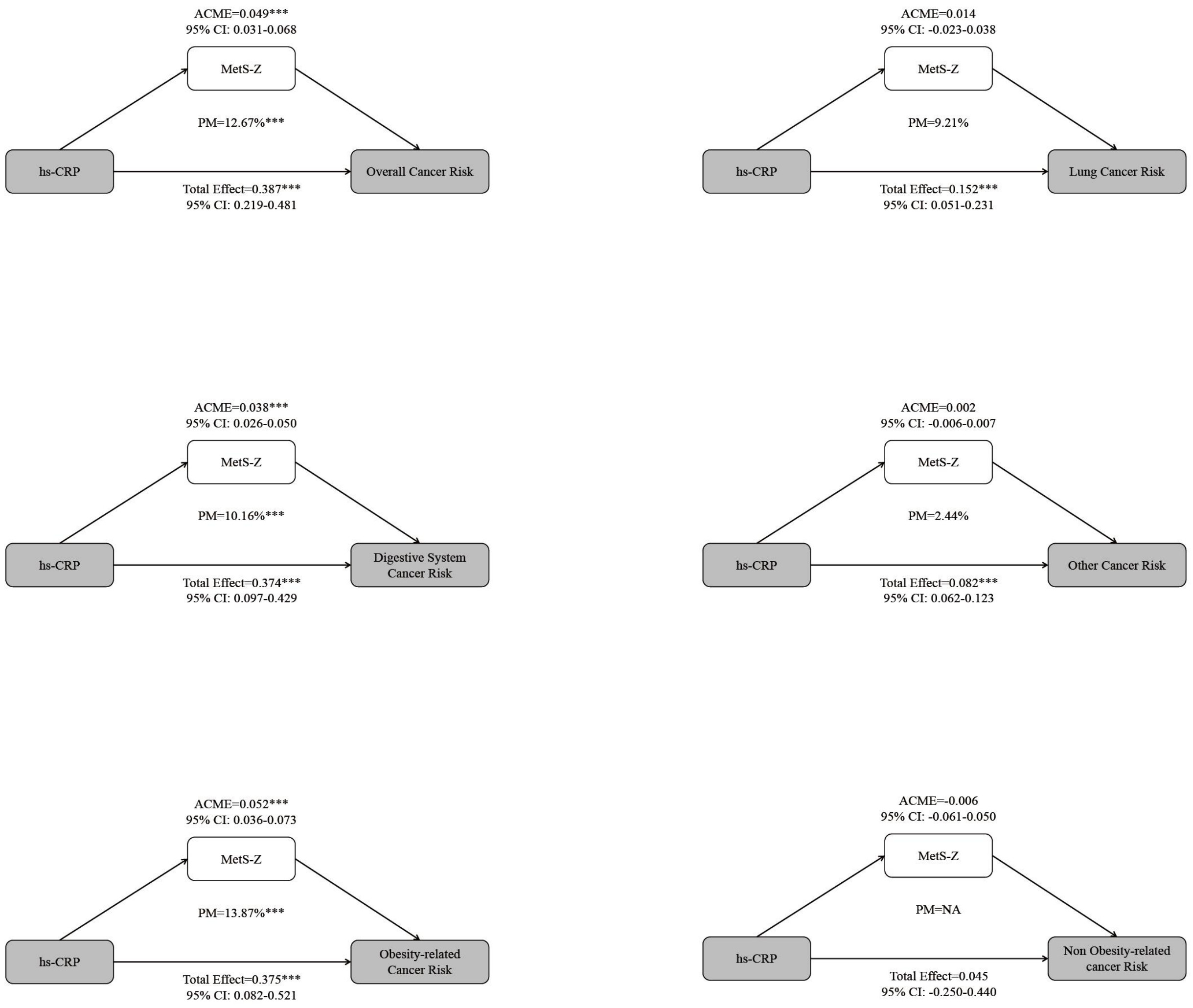

Table S1. Value of Average CumMetS-Z, mean MetS-Z, Average CumCRP, and mean hsCRP during the exposure period

	Mean (SD)	MAX	MIN	P25	P50	P75
Average CumMetS-Z[a]	-0.16(0.78)	6.86	-3.31	-0.64	-0.21	0.25
mMetS-Z [b]	-0.16(0.88)	6.01	-3.35	-0.71	-0.22	0.31
Average CumCRP [c]	2.86(4.51)	64.87	0.07	0.86	1.60	3.18
mCRP [d]	2.70(6.34)	101	0.01	0.5	1.15	2.84

[a] Average CumMetS-Z: average cumulative atherogenic index of plasma in the exposure period.

[b] mMetS-Z: mean value of MetS-Z in the three transient measures in the exposure period.

[c] Average CumCRP: average cumulative high-sensitivity C-reactive protein in the exposure period.

[d] mCRP: mean value of hs-CRP in the three transient measures in the exposure period.

Table S2 Definition of covariates

Covariates	Definition
Regular physical activity	≥ 3 times/week, ≥ 30 minutes/time of physical exercise is considered regular physical exercise
Current smoker	Smoking was defined as having 1 cigarette/day at least for more than 6 months.
Current drinker	Alcohol consumer was defined as having drunk ≥100 mL/day of alcohol lasting for more than 6 months, regardless of the type of alcohol.
Hypertension	Systolic blood pressure ≥ 140 mm Hg, and/or diastolic blood pressure ≥ 90 mm Hg, and/or previously diagnosed with hypertension.
Diabetes mellitus	Fasting blood glucose level ≥ 7.0mmoL/L, taking oral hypoglycemic drugs or insulin, or having a self-reported medical history.
Fatty liver	The ultrasonic examination (ACUSON X300, Siemens, Germany) was used to examine the abdominal region, including liver, gallbladder, pancreas and spleen of each participant after fasting for at least 8 hours by a panel of specialists. Fatty liver was diagnosed by abdominal ultrasonography according to previous clinically established criteria [3] or through medical records from the Tangshan Medical Insurance System
Medication Use	The medication use records were all from the questionnaire, for example, are you taking antihypertensive, hypoglycemic, or lipid-lowering drugs? Do you follow medical advice and take your medication regularly? All patients who regularly take medication are recorded as' yes'.
Laboratory Testing	All participants underwent at least 8 hours of fasting and then received blood tests in the designated hospital, which were uniformly. All the serum samples were analyzed by an auto-analyzer (Hitachi 747; Hitachi, Tokyo, Japan) at the central laboratory of Kailuan General Hospital.
High-fat diet	All participants were asked during the survey if they frequently consume high-fat diet?

Table S3 Hazard ratio (95% CI) of overall cancer risk for Average CumCRP stratifed by Average CumMetS-Z

	Average CumCRP, HRs (95% CIs)							
	G1	G2		G3	P	P for trend	per SD	P
**Overall**								
Event/Total	564/13848	978/19303		762/12022				
IR[a]	3.82	4.85		6.31				
Model 1	Ref.	1.27(1.14,1.41)	<0.001	1.65(1.48,1.84)	<0.001	<0.001	1.03(1.01,1.05)	<0.001
Model 2	Ref.	1.19(1.07,1.33)	<0.001	1.39(1.24,1.56)	<0.001	<0.001	1.04(1.02,1.06)	<0.001
**Average CumMetS-Z T1**								
Events/Total	237/5965	290/5785		213/3307				
IR[a]	3.73	4.78		6.42				
Model 1	Ref.	1.28(1.08,1.52)	0.005	1.72(1.43,2.07)	<0.001	<0.001	1.10(1.05,1.15)	<0.001
Model 2	Ref.	1.20(1.01,1.42)	0.043	1.42(1.17,1.72)	<0.001	<0.001	1.06(1.01,1.11)	0.010
**Average CumMetS-Z T2**								
Event/Total	189/4664	339/6718		239/3674				
IR[a]	3.79	4.81		6.42				
Model 1	Ref.	1.27(1.06,1.51)	0.009	1.69(1.4,2.05)	<0.001	<0.001	1.05(1.02,1.09)	0.005
Model 2	Ref.	1.22(1.02,1.45)	0.032	1.51(1.24,1.84)	<0.001	<0.001	1.04(1.01,1.08)	0.027
**Average CumMetS-Z T3**								
Event/Total	138/3219	349/6800		310/5041				
IR[a]	4.06	4.97		6.17				
Model 1	Ref.	1.34(1.10,1.64)	0.004	1.70(1.38,2.08)	<0.001	<0.001	1.09(1.03,1.15)	0.003
Model 2	Ref.	1.26(1.03,1.54)	0.027	1.44(1.16,1.77)	<0.001	0.008	1.07(1.01,1.12)	0.030
**Average CumMetS-Z Low**								
Event/Total	401/9653	325/6606		383/6327				
IR[a]	3.90	4.69		5.94				
Model 1	Ref.	1.16(1,1.34)	0.467	1.51(1.21,1.88)	<0.001	<0.001	1.05(1.03,1.08)	<0.001
Model 2	Ref.	1.09 (0.86,1.38)	0.051	1.34(1.15,1.56)	<0.001	<0.001	1.05(1.01,1.09)	0.014
**Average CumMetS-Z High**								
Event/Total	282/8713	387/7636		526/8713				
IR[a]	3.21	4.88		5.98				
Model 1	Ref.	1.14(0.98,1.33)	0.097	1.40(1.21,1.61)	<0.001	<0.001	1.05(1.03,1.08)	<0.001
Model 2	Ref.	1.11(0.95,1.29)	0.203	1.26(1.07,1.48)	0.005	0.005	1.05(1.01,1.08)	0.009

[a]The incidence rate (IR) is per 1000 person-years.

Model 1 was crude model.

Model 2 was adjusted for age, sex, education, marital status, smoking, drinking, physical activities, sedentary, family history of cancer, BMI, hypertension, diabetes, fatty liver, antihypertensives, hypoglycemic drugs, lipid-lowering drugs, waist circumference, HDL, SBP, TG, and FBG.

G1: Average CumCRP < 1mg/L; G2: 1mg/L ≤ Average CumCRP < 3mg/L; G3: Average CumCRP ≥ 3mg/L. Average CumMetS-Z T1: -3.31 to -0.48. Average CumMetS-Z T2: -0.48 to 0.07. Average CumMetS-Z T3: 0.07 to 6.86. Average CumMetS-Z Low: -3.31 to -0.21. Average CumMetS-Z High: -0.21 to 6.86.

Table S4 Subgroup analysis

	Event/Total	G1	G2	G3	G4	G5	G6	P for interaction
**Sex**								
men	1805/35684	Ref.	1.16(0.95,1.42)	1.31(1.12,1.55)	1.22(1.02,1.46)	1.48(1.23,1.78)	1.43(1.18,1.74)	0.917
women	499/9489	Ref.	1.25(0.81,0.9)	1.07(0.8,1.43)	1.47(1.06,2.03)	1.08(0.75,1.55)	1.71(1.20,2.44)	
**Age (years)**								**0.001**
<60	1649/37517	Ref.	1.26(1.03,1.54)	1.29(1.09,1.51)	1.35(1.13,1.61)	1.49(1.22,1.81)	1.72(1.41,2.10)	
≥60	655/7656	Ref.	0.85(0.56,1.29)	1.07(0.79,1.44)	0.95(0.69,1.32)	1.08(0.79,1.47)	0.95(0.68,1.33)	
**Smoke**								**0.039**
no	1505/29761	Ref.	1.16(0.92,1.46)	1.29(1.08,1.56)	1.44(1.18,1.75)	1.48(1.2,1.82)	1.53(1.23,1.89)	
yes	799/15412	Ref.	1.14(0.85,1.51)	1.2(0.96,1.5)	0.96(0.74,1.24)	1.26(0.96,1.66)	1.39(1.05,1.85)	
**Alcohol use**								0.210
no	1525/28270	Ref.	1.14(0.9,1.43)	1.26(1.05,1.51)	1.38(1.13,1.68)	1.53(1.25,1.87)	1.52(1.23,1.88)	
yes	779/16903	Ref.	1.18(0.89,1.58)	1.26(1,1.58)	1.06(0.81,1.38)	1.18(0.88,1.58)	1.43(1.07,1.91)	
**Fatty liver**								**0.001**
no	1499/30482	Ref.	1.13(0.91,1.4)	1.31(1.12,1.53)	1.25(1.03,1.52)	1.45(1.21,1.75)	1.74(1.41,2.14)	
yes	805/14691	Ref.	0.91(0.64,1.29)	0.93(0.66,1.31)	0.93(0.68,1.28)	1.05(0.73,1.52)	0.96(0.69,1.34)	
**Obesity-BMI**								0.907
no	1844/36609	Ref.	1.13(0.93,1.38)	1.31(1.11,1.53)	1.27(1.07,1.51)	1.35(1.12,1.62)	1.49(1.24,1.8)	
yes	460/8564	Ref.	1.2(0.81,1.78)	1.06(0.77,1.47)	1.16(0.82,1.65)	1.67(1.16,2.39)	1.47(1,2.17)	
**Obesity-WC**								0.435
no	718/16347	Ref.	1.11(0.82,1.51)	1.32(1.08,1.61)	1.21(0.92,1.6)	1.21(0.93,1.58)	1.14(0.78,1.65)	
yes	1586/28826	Ref.	1.14(0.91,1.44)	1.18(0.96,1.46)	1.24(1.01,1.52)	1.47(1.18,1.83)	1.53(1.24,1.89)	
**Physical activity**								0.097
no	1884/38891	Ref.	1.14(0.94,1.39)	1.27(1.09,1.49)	1.25(1.05,1.49)	1.46(1.22,1.75)	1.56(1.29,1.88)	
yes	420/6282	Ref.	1.14(0.75,1.75)	1.18(0.85,1.65)	1.18(0.82,1.69)	1.12(0.75,1.68)	1.17(0.79,1.73)	
**Hypertension**								0.402
no	1981/40340	Ref.	1.11(0.92,1.35)	1.27(1.1,1.47)	1.24(1.05,1.46)	1.42(1.19,1.68)	1.43(1.19,1.71)	
yes	323/4833	Ref.	1.27(0.73,2.21)	1.07(0.64,1.79)	1.17(0.72,1.9)	1.17(0.66,2.05)	1.56(0.94,2.58)	
**Diabetes**								**0.019**
no	2074/41362	Ref.	1.12(0.94,1.35)	1.26(1.09,1.45)	1.23(1.05,1.44)	1.42(1.21,1.68)	1.51(1.27,1.80)	
yes	230/3811	Ref.	1.04(0.36,1.66)	1.08(0.44,2.61)	0.98(0.48,2.01)	1.27(0.53,3.06)	0.97(0.46,2.04)	

**Dyslipidemia**								0.189
no	1579/30458	Ref.	1.18(0.95,1.48)	1.27(1.09,1.47)	1.16(0.96,1.4)	1.38(1.16,1.64)	1.49(1.21,1.83)	
yes	725/14715	Ref.	1.3(0.82,2.07)	1.17(0.7,1.97)	1.57(1.01,2.45)	1.45(0.83,2.51)	1.79(1.13,2.84)	

G1: average cumMetS-Z < -0.21 and average cumCRP < 1mg/L; G2: average cumMetS-Z ⩾ -0.21 and average cumCRP < 1mg/L; G3: average cumMetS-Z < -0.21 and 1mg/L ⩽ average cumCRP < 3mg/L; G4: average cumMetS-Z ⩾ -0.21 and 1mg/L ⩽ average cumCRP < 3mg/L; G5: average cumMetS-Z < -0.21 and average cumCRP ⩾ 3mg/L; G6: average cumMetS-Z ⩾ -0.21 and average cumCRP ⩾ 3mg/L.

Model was adjusted for age, sex, education, marital status, smoking, drinking, physical activities, sedentary, family history of cancer, BMI, hypertension, diabetes, fatty liver, antihypertensives, hypoglycemic drugs, lipid-lowering drugs, waist circumference, HDL, SBP, TG, and FBG.

Table S5 Hazard ratios (HRs) for cancer risk upon co-exposure stratifed by baseline hsCRP thresholds (1, 3 mg/L) and baseline MetS-Z (median)

	G1	G2		G3		G4		G5		G6	
Overall cancer		HR (95%CI)	P	HR (95%CI)	P	HR (95%CI)	P	HR (95%CI)	P	HR (95%CI)	P
Model 1	Ref.	1.01(0.87,1.18)	0.868	1.14(0.98.1.32)	0.086	1.24(1.07,1.42)	0.003	1.45(1.27,1.65)	<0.001	1.52(1.32,1.75)	<0.001
Model 2	Ref.	0.95(0.81,1.11)	0.523	1.10(0.95,1.27)	0.208	1.14(0.98,1.32)	0.092	1.21(1.05,1.40)	0.009	1.36(1.18,1.56)	<0.001
Model 3	Ref.	1.01(0.85,1.20)	0.880	1.10(0.95,1.28)	0.197	1.16(1,1.37)	0.050	1.23(1.04,1.45)	0.017	1.34(1.16,1.55)	<0.001
Obesity-related cancer[a]											
Model 1	Ref.	1.03(0.87,1.22)	0.754	1.14(0.97,1.34)	0.122	1.31(1.12,1.52)	<0.001	1.50(1.29,1.75)	<0.001	1.53(1.33,1.77)	<0.001
Model 2	Ref.	1(0.84,1.19)	0.977	1.09(0.93,1.29)	0.296	1.19(1.01,1.40)	0.035	1.27(1.09,1.49)	0.003	1.32(1.13,1.54)	<0.001
Model 3	Ref.	1.05(0.87,1.27)	0.612	1.10(0.93,1.29)	0.270	1.26(1.05,1.51)	0.013	1.30 (1.10, 1.53)	0.002	1.33(1.10,1.59)	0.003
Non obesity-related cancer											
Model 1	Ref.	0.95(0.67,1.36)	0.790	1.13(0.81.1.58)	0.456	0.94(0.67,1.32)	0.735	1.10(0.80,1.51)	0.563	1.60(1.18,2.18)	0.003
Model 2	Ref.	0.96(0.67,1.38)	0.827	1.12(0.81.1.57)	0.490	0.90(0.62,1.29)	0.556	0.95(0.67,1.35)	0.784	1.52(1.11,2.08)	0.009
Model 3	Ref.	0.86(0.58,1.29)	0.472	1.13(0.81,1.57)	0.484	0.80(0.54,1.20)	0.283	0.84(0.56,1.26)	0.396	1.52(1.09,2.10)	0.013
Digestive system cancer[b]											
Model 1	Ref.	1.28(0.99,1.66)	0.061	1.39(1.08,1.79)	0.010	1.60(1.26,2.04)	<0.001	1.40(1.10,1.78)	0.006	1.78(1.40,2.27)	<0.001
Model 2	Ref.	1.19(0.91,1.55)	0.205	1.34(1.04,1.73)	0.024	1.44(1.12,1.86)	0.005	1.21(0.93,1.57)	0.148	1.58(1.24,2.02)	<0.001
Model 3	Ref.	1.30(0.97,1.74)	0.083	1.36(1.05,1.75)	0.018	1.59(1.20,2.10)	<0.001	1.34(1,1.82)	0.044	1.59(1.23,2.07)	<0.001
Lung cancer											
Model 1	Ref.	0.83(0.62,1.12)	0.229	1.03(0.78,1.36)	0.828	1.01(0.77,1.32)	0.952	1.29(0.99,1.68)	0.061	1.28(1,1.64)	0.050
Model 2	Ref.	0.82(0.60,1.11)	0.199	1(0.76.1.33)	0.982	0.95(0.71,1.27)	0.749	1.15(0.88,1.51)	0.301	1.16(0.88,1.52)	0.293
Model 3	Ref.	0.81(0.58,1.14)	0.232	1.01(0.76,1.33)	0.970	0.94(0.68,1.30)	0.72	1.13(0.85,1.49)	0.393	1.11(0.81,1.53)	0.514
Other cancer											
Model 1	Ref.	0.94(0.74,1.21)	0.646	1.02(0.81,1.30)	0.848	1.13(0.90,1.41)	0.299	1.50 (1.20,1.86)	<0.001	1.60(1.31,1.96)	<0.001
Model 2	Ref.	0.97(0.76,1.25)	0.834	0.99(0.78,1.25)	0.906	1.05(0.83,1.33)	0.694	1.26(1,1.58)	0.048	1.34(1.08,1.67)	0.009
Model 3	Ref.	0.97(0.73,1.27)	0.798	0.99(0.78,1.25)	0.917	1.04(0.80,1.35)	0.765	1.22(0.94,1.58)	0.131	1.32(1.05,1.66)	0.018

G1: average cumMetS-Z < -0.22 and average cumCRP < 1mg/L; G2: average cumMetS-Z ⩾ -0.22 and average cumCRP < 1mg/L; G3: average cumMetS-Z < -0.22 and 1mg/L ⩽ average cumCRP < 3mg/L; G4: average cumMetS-Z ⩾ -0.22 and 1mg/L ⩽ average cumCRP < 3mg/L; G5: average cumMetS-Z < -0.22 and average cumCRP ⩾ 3mg/L; G6: average cumMetS-Z ⩾ -0.22 and average cumCRP ⩾ 3mg/L.

Model 1 was crude model. Model 2 was adjusted for age, sex, education, marital status, smoking, drinking, physical activities, sedentary, family history of cancer, BMI, hypertension, diabetes, fatty liver. Model 3 was adjusted for model 2 and antihypertensives, hypoglycemic drugs, lipid-lowering drugs, waist circumference, HDL, SBP, TG, and FBG.

[a] Obesity-related cancer include esophagus, stomach, colon, rectum, liver, pancreas, lung, malignant melanoma, breast, corpus uteri, ovaries, prostate, kidney, bladder, brain and lymphoid and hematopoietic cancer.

[b] Digestive system cancer include esophagus, stomach, small intestine, colon, rectum, liver, pancreas cancer, and bile and extrahepatic cholangiocarcinoma.

Table S6 Sensitivity analyses

	G1	G2	G3	G4	G5	G6
		HR (95%CI)	HR (95%CI)	HR (95%CI)	HR (95%CI)	HR (95%CI)
**Exclude events in the first follow-up visit (N=44904)**	Ref.	1.17(0.97,1.41)	1.28(1.10,1.49)	1.28(1.08,1.51)	1.36(1.14,1.62)	1.47(1.23,1.76)
**Excluding participants receiving hypoglycemic medication treatment (N=44271)**	Ref.	1.12(0.93,1.34)	1.25(1.09,1.45)	1.22(1.04,1.43)	1.44(1.21,1.70)	1.50(1.26,1.79)
**Excluding participants receiving lipid-lowering medication treatment (N=44807)**	Ref.	1.15(0.97,1.38)	1.26(1.09,1.45)	1.26(1.08,1.48)	1.38(1.17,1.63)	1.47(1.24,1.74)
**Excluding participants receiving antihypertensive medication treatment (N=39141)**	Ref.	1.11(0.92,1.34)	1.23(1.04,1.45)	1.27(1.09,1.47)	1.43(1.20,1.71)	1.42(1.18,1.71)
**Excluding participants who typically follow a high-fat diet pattern (N=41203)**	Ref.	1.17(0.97,1.42)	1.23(1.06,1.43)	1.26(1.07,1.48)	1.41(1.19,1.67)	1.43(1.20,1.71)
**Excluding participants with a family history of cancer (N=42514)**	Ref.	1.14(0.95,1.37)	1.22(1.06,1.41)	1.22(1.04,1.43)	1.40(1.18,1.65)	1.47(1.24,1.75)
**Excluding participants with hs-CRP > 10mg /L (N=43443)**	Ref.	1.14(0.95,1.37)	1.25(1.08,1.44)	1.24(1.06,1.45)	1.38(1.15,1.66)	1.46(1.22,1.76)
**Adjusting for time-varying covariates**						
Model 1*	Ref.	1.15(0.98,1.37)	1.32(1.17,1.55)	1.36(1.20,1.59)	1.68(1.45,1.98)	1.82(1.60,2.13)
Model 2[#]	Ref.	1.17(0.99,1.39)	1.36(1.18,1.57)	1.41(1.22,1.62)	1.77(1.51,2.06)	1.93(1.67,2.24)

G1: average cumMetS-Z < -0.21 and average cumCRP < 1mg/L; G2: average cumMetS-Z ⩾ -0.21 and average cumCRP < 1mg/L; G3: average cumMetS-Z < -0.21 and 1mg/L ⩽ average cumCRP < 3mg/L; G4: average cumMetS-Z ⩾ -0.21 and 1mg/L ⩽ average cumCRP < 3mg/L; G5: average cumMetS-Z < -0.21 and average cumCRP ⩾ 3mg/L; G6: average cumMetS-Z ⩾ -0.21 and average cumCRP ⩾ 3mg/L.

*Model 1 was adjusted for age (time-varying), sex (time-varying), education (time-varying), marital status (time-varying), smoking (time-varying), drinking (time-varying), physical activities (time-varying), sedentary (time-varying), family history of cancer (time-varying), BMI (time-varying), hypertension (time-varying), diabetes (time-varying), fatty liver (time-varying).

[#]Model 2 was adjusted for model 2 and antihypertensives (time-varying), hypoglycemic drugs (time-varying), lipid-lowering drugs (time-varying), waist circumference (time-varying), HDL (time-varying), SBP (time-varying), TG (time-varying), and FBG (time-varying).

Reference

[1] Gurka MJ, Filipp SL, Musani SK et al. Use of BMI as the marker of adiposity in a metabolic syndrome severity score: Derivation and validation in predicting long-term disease outcomes. Metabolism 2018; 83: 68-74.

[2] Executive Summary of The Third Report of The National Cholesterol Education Program (NCEP) Expert Panel on Detection, Evaluation, And Treatment of High Blood Cholesterol In Adults (Adult Treatment Panel III). Jama 2001; 285: 2486-2497.

[3] Esterson YB, Grimaldi GM. Radiologic Imaging in Nonalcoholic Fatty Liver Disease and Nonalcoholic Steatohepatitis. Clin Liver Dis. 2018;22(1):93-108.
